# A CNN-based misleading video detection model

**DOI:** 10.1038/s41598-022-10117-y

**Published:** 2022-04-12

**Authors:** Xiaojun Li, Xvhao Xiao, Jia Li, Changhua Hu, Junping Yao, Shaochen Li

**Affiliations:** 1 Xi’an Research Institute of High-Tech, Xi’an, 710025 China; 2 School of Business, East China University of Science and Technology, Shanghai, 200237 China

**Keywords:** Electrical and electronic engineering, Information theory and computation

## Abstract

Videos, especially short videos, have become an increasingly important source of information in these years. However, many videos spread on video sharing platforms are misleading, which have negative social impacts. Therefore, it is necessary to find methods to automatically identify misleading videos. In this paper, three categories of features (content features, uploader features and environment features) are proposed to construct a convolutional neural network (CNN) for misleading video detection. The experiment showed that all the three proposed categories of features play a vital role in detecting misleading videos. Our proposed approach that combines three categories of features achieved the best performance with the accuracy of 0.90 and the F1 score of 0.89. It also outperformed other baselines such as SVM, k-NN, decision tree and random forest models by more than 22%.

## Introduction

Online videos on media sharing platforms have risen to be a dominating source of information. However, as misleading videos grow rife on social media, the information from these videos will considerably mislead the audience, or have far-reaching negative impacts on the society. According to a report from the United Nations, during the outbreak of Covid-19 in late 2019, more than a quarter of videos about this epidemic on YouTube platform contain misleading information^1^. This would seriously hinder epidemic prevention. The main purpose of making misleading videos is to obtain commercial benefits (such as cajoling users to click phishing links or buy products through false advertising). Therefore, driven by commercial interests, misleading videos have increased rapidly on social media. For example, during the epidemic in 2020, Facebook deleted 16 million pieces of false contents (including texts, images, and videos) and issued 167 million information warnings^2^. From the outbreak of the epidemic to August 2021, YouTube deleted more than 1 million misleading videos^3^. The excessive number of misleading videos on the media sharing platforms makes it a challenge to achieve automatic detection of these videos.

Misleading videos are different from fake videos on the media sharing platforms^4^. For misleading videos, the footage itself may be real, in the sense that it’s showing something that really happened, but is mislabeled to make a political point, or get shared^4^. Videos like this might say they were filmed in one country, when they originate from another, or incorrectly name the people involved. Fake videos are those that aren’t real, either because they’ve been staged or digitally doctored^4^. The video generated with deepfake^5^ in which a person in an existing image or video is replaced with someone else's likeness is an example of fake videos. We focus on the detection of misleading videos in this study.

Although the detection of fake videos has received a lot of attention in recent years^6,7^, the research on the detection of misleading videos is still lacking. Since fake videos are synthetic videos in nature, the detection approaches are mainly based on the computer vision technology such as tampering detection^8^, copy-move forgery detection^9^ and motion magnification detection^10^. As a result, they cannot be applied for the misleading videos directly. The most relevant works related to the detection of misleading videos focus on false news^11,12^, fake reviews^13–15^, spam content^16,17^ and tampered videos^18,19^. However, the false news or reviews are presented in the format of text; videos, however, involve various formats of contents, and are hence more difficult to analyze than texts. In addition, misleading video is usually deliberately made, with the theme kept highly consistent with the content. Therefore, misleading video detection is totally different from detection of spam (e.g., advertising). In short, it is not feasible to directly apply the existing detection approaches for false news, spam content, and tampered videos to detection of misleading videos on media sharing platforms. As a result, it is urgent to develop a new method to automatically detect misleading videos.

In this paper, we propose three categories of features for misleading video detection—content features, uploader features, and environment features. To the best of our knowledge, this is the first study that incorporates all the three categories of features in the same detection approach. Furthermore, we propose a CNN-based classifier to detect misleading videos based on these categories of features.

## Related work

A review of literature suggests that existing works on false information detection mainly focus on fake news detection, fake review detection and deepfake detection.

### Fake news detection

There are mainly two types of approaches for fake news detection: the traditional feature-based approach, and the deep learning-based approach.

The feature-based approach detects fake videos by identifying important features, such as text-based features (e.g., emotional polarity, modal particles, writing style), knowledge-based features (i.e., manual verification of facts), communication-based features (i.e., transmission mode of information in the network), and source-based features (i.e., reliability of information source). For example, Castillo et al.^11^ extracted four types of features, i.e., content features, user features, subject features, and dissemination features, to detect false news and assess news reliability. Yang et al.^20^ applied the application features and location features to improve the accuracy of false news detection on Sina Weibo. Zhao et al.^21^ proposed an approach for early detection of rumors on social media from enquiry posts. This approach tries to find signature text phrases that are used by a few people to express skepticism about factual claims and are rarely used to express anything else, which can be used as indicators for rumor clusters. Kwon et al.^22^ constructed a time series model with time features, language characteristics features and communication structure features to detect the falsity of information.

In recent years, deep learning has seen increased adoption in the development of fake news detection algorithms. The deep learning approach optimizes and transforms the model according to the characteristics of the data itself. For example, Ma et al.^12^ used the time-varying context information of RNN learning information to distinguish the falsity of information. Yu et al.^23^ used a CNN to mine key features of input sequences for early detection of false information.

### Fake review detection

The research on fake review detection mainly focuses on three streams of approaches: the content-based approach, the non-content-based approach and the spammer approach.

The content-based approach detects fake reviews based on content features such as n-grams, keywords, or knowledge embedded in the text. For example, Ahmed et al.^13^ introduced a new n-gram model to automatically detect fake contents with a particular focus on fake reviews. Levchuk et al.^24^ described a model for detecting conflicts in multi-source textual knowledge. The model constructs semantic graphs representing patterns of multi-source knowledge conflicts and anomalies, and detects these conflicts by matching pattern graphs against the data graph constructed by soft co-reference between entities and events in multiple sources. Zhang et al.^25^ proposed a novel truth discovery method, named “TextTruth”, which jointly groups the keywords extracted from the answers of a specific question into multiple interpretable factors, and infers the trustworthiness of both answer factors and answer providers.

The non-content-based approach identifies fake reviews based on other non-content cues such as the rating score distribution or temporal patterns. For example, Akoglu et al.^14^ represented the review dataset as a bipartite network, based on which they proposed a framework called FRAAUDEAGLE for false review detection using the network effect between reviewers and products. Xie et al.^26^ proposed an approach to detect review spams by identifying unusually correlated temporal patterns. They found that the normal reviewers’ arrival pattern is stable and uncorrelated to their rating pattern temporally. In contrast, spam attacks are usually bursty, either positively or negatively correlated to the rating.

The spammer approach identifies review spammers. For example, Hu et al.^15^ found that the emotional cues are important to tell spammers from normal users. Wu et al.^27^ proposed a new sparse group modeling method to describe social networks, and combined with the sparse group modeling for adaptative spammer detection (SGASD) framework to detect spammers. Yusof et al.^28^ proposed a new set of features for detection of malicious users by constructing features based on the EdgeRank algorithm. Bhat et al.^29^ proposed a community-based framework that uses user characteristics to identify spammers in online social networks. Mukherjee et al.^30^ put forward an unsupervised author space model, through which all kinds of behavioral footprints of reviewers are obtained, and then the false reviewers are detected.

### Deepfake detection

Deepfakes leverage powerful techniques from machine learning and artificial intelligence to manipulate or generate visual and audio content with a high potential to deceive. There are mainly two types of methods for detection of deepfakes: the image-based approach and the video-based approach.

The image-based approach works by detecting forgery of static images. For example, Yang et al.^18^ proposed a generalized model for small-size recapture image forensics based on Laplacian convolutional neural networks (CNNs). Different from other CNN models, they put the signal enhancement layer into the CNN structure and a Laplacian filter is used in the signal enhancement layer. Bayar et al.^31^ developed a new form of convolutional layer that is specially designed to suppress an image’s content and adaptively learn manipulation detection features. Their proposed approach can automatically learn to detect multiple image manipulations without relying on pre-selected features or any preprocessing. Hsu et al.^32^ proposed a deep learning-based approach that detects fake images using the contrastive loss. Specifically, the reduced DenseNet is developed to a two-streamed network structure to allow pairwise information as the input. Then, the proposed common fake feature network is trained using the pairwise learning to distinguish the features between the fake and real images.

The video-based approach works by investigating the temporal characteristics of continuous frames. Amerini et al.^19^ introduced a new technique that distinguishes synthetic generated portrait videos from natural ones by exploiting inconsistencies due to the prediction error in the re-encoding phase. They applied a long short-term memory (LSTM) model network to learn the temporal correlation among consecutive frames. Sabir et al.^33^ proposed the best strategy for combining variations in CNN-based image manipulation detection models along with domain-specific face preprocessing techniques through extensive experimentation to obtain state-of-the-art performance on publicly available video-based facial manipulation benchmarks. Güera et al.^34^ proposed a temporal-aware pipeline to automatically detect deepfake videos. Their system uses a CNN to extract frame-level features, which are then used to train a recurrent neural network (RNN) that learns to classify whether a video has been manipulated or not.

In summary, misleading videos are mislabelled real videos rather than synthesized videos, techniques for detection of deepfakes are not appropriate for detection of misleading videos. In addition, existing research on false content detection mainly depends on content features and uploader features. However, environment features are rarely used in detection. Media sharing platforms usually provide environment functions (e.g., thumbs-up and -down, favorites, forwarding, etc.). These environment features are important sources to identify misleading videos. In addition, the emotion embedded in the content is rarely used as a feature for misleading video detection. The emotion in a misleading video is often stronger than that in a normal video, so it could be used as an important indicator for misleading videos. To bridge the research gap identified above, we combined the three types of features (content features, uploader features and environment features) and constructed a CNN model. The model can learn higher-level potential relationships between features to detect misleading videos.

## Methodology and model

In this study, we first extract three categories of features from videos and then build a CNN-based detection algorithm.

### Video feature extraction

#### Content features

Content features are derived from the video content. All audio information were converted into texts before feature extraction. In this study, we include four types of content features, i.e., sentiment polarity, the number of modal particles, the number of personal pronouns, and text length.

1. Sentiment polarity (C-Sen-Po). Misleading videos usually contain strong emotions to compel viewers to believe the false information in the video. Therefore, sentiment polarity is a vital measure for detection of misleading videos.
  Sentiment polarity is calculated by the steps described in Fig. 1. First, particles and stop words are eliminated in a text; second, sentiment words and their qualifiers are detected in the text; third, the sentiment polarity of the text is rated through aggregate calculation of sentiment words and qualifiers.

**Figure 1:**
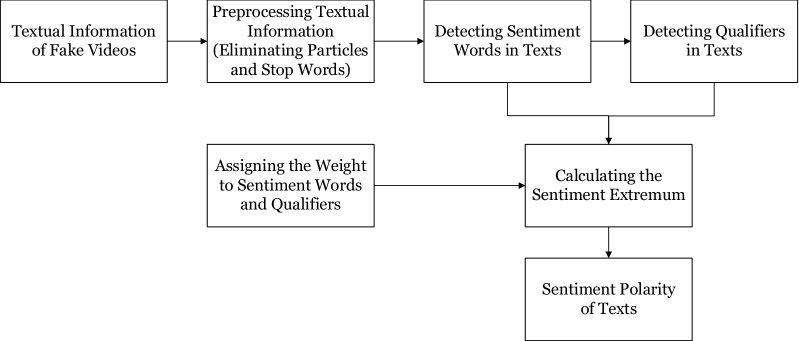
Process of extracting sentiment polarity features in texts.
  In this study, positive and negative emotions are divided into six levels (scored 1–6 from the weakest to the strongest) according to the sentiment lexicon of Hownet. In the sentiment lexicon of Hownet, each word falls into one of the twelve levels mentioned above and is assigned a sentiment score. The sentiment polarity is calculated by summing up the sentiment score of each word ${ws}_{i}$ in the text:
  1 $$\begin{array}{c}Sentiment=\sum {ws}_{i},\end{array}$$
2. The number of modal particles (C-Num-MoPar). Aside from words that express strong feelings, intensive emotions can be conveyed to viewers through modal particles. The number of modal particles in a text can be obtained through the detection and aggregation of modal particles in the textual content of a video, and are given by:
  2 $$\begin{array}{c}SumTone= \sum_{i=1}^{n}tone\left({w}_{i}\right),\end{array}$$
  where $SumTone$ is the number of modal particles; $tone\left({w}_{i}\right)$ determines whether a word is a modal particle or not; and ${w}_{i}$ denotes the $i$-th word in the text.
3. The number of personal pronouns (C-Num-PerPro). Observations show that third-person pronouns are more often used than first-person pronouns in disinformation^35^. Therefore, we construct a new feature as the number of personal pronouns, which can be attained through the detection and aggregation of these pronouns in the textual content of a video, and are given by:
  3 $$\begin{array}{c}SumPer= \frac{\sum_{i=1}^{n}TPerspro\left({w}_{i}\right)-\sum_{i=1}^{n}FPerspro\left({w}_{i}\right)}{\sum_{i=1}^{n}TPerspro\left({w}_{i}\right)+\sum_{i=1}^{n}FPerspro\left({w}_{i}\right)},\end{array}$$
  where $SumPer$ refers to the percentage of personal pronouns in the total words of the text; $FPerspro\left({w}_{i}\right)$ judges whether a word is a first-person pronoun or not; $TPerspro\left({w}_{i}\right)$ determines whether a word is a third-person pronoun or not; and ${w}_{i}$ denotes the word in the $i$-th place in the text.
4. According to the study by Day et al.^36^, the text length of false information also serves as an effective indicator for falsehood. Unlike regular information, the false content is either too short or extremely long in length. Video text length (C-Vtext-Len) can be obtained through the aggregation of words in a text, and is expression as:
  4 $$\begin{array}{c}SumWord= \sum {w}_{i},\end{array}$$
  where $SumWord$ represents the total number of words in a text; and ${w}_{i}$ indicates the word in the $i$-th place in the text.

#### Uploader features

Studies on detection of spam mails and fake comments showed that these unwanted messages could be spotted by observing the message sender^37^. In this study, we propose four uploader features, namely the follower-following ratio, the number of likes received, the date of most recent upload, and the number of total views.

1. The follower-following ratio (FF-R) is the ratio of the number of an uploader’s followers to the sum of the uploader’s followers and the accounts that the uploader follows:
  5 $$\begin{array}{c}{Fol}_{F}= \frac{{FF}_{Fans}}{{FF}_{Attention}+{FF}_{Fans}},\end{array}$$
  where ${Fol}_{F}$ refers to the follower-following ratio; ${FF}_{Fans}$ represents the number of followers a video uploader has; ${FF}_{Attention}$ denotes the number of other video accounts this video uploader follows.
2. The number of likes a video uploader receives (Num-Likes) means all the likes the uploader has gained from other users. The information is accessible on the uploader’s profile page.
3. The date of most recent upload (Re-upload) is the most recent date when an uploader publishes a video on the platform. This feature indicates how active the uploader is.
4. The number of total views (Num-ToVi) is the times the uploader’s videos being played by other users on the platform, which is expressed as follows:
  6 $$\begin{array}{c}SumPlay= \sum_{i=1}^{n}{Play}_{i},\end{array}$$
  where $SumPlay$ denotes the times that an uploader’s videos has been played; ${Play}_{i}$ means the views of the video in the $i$-th place; and $n$ represents the number of videos uploaded.

#### Environment features

In this study, the environment features of a video include the number of likes, forwards, favorites and rewards. In addition, we use the sentiment polarity of the top three popular comments to present the impact of a video on viewers as the environment features. Similar to the content features, we also consider the sentiment polarity (E-Sen-Po), the number of modal particles (E-Num-MoPar), the number of personal pronouns (E-Num-PerPro) and text length (E-Vtext-Len) in each comment.

### The CNN-based false information detection model

Spotting a misleading video would ultimately require falsehood detection of the video clip, and in this paper, we adopted a CNN-based model for misleading video detection. Compared with other neural network-based models that import datasets into the network for training, the proposed model trains the network on video features, allowing the neural network to learn the features and associations between these features.

**Figure 2:**
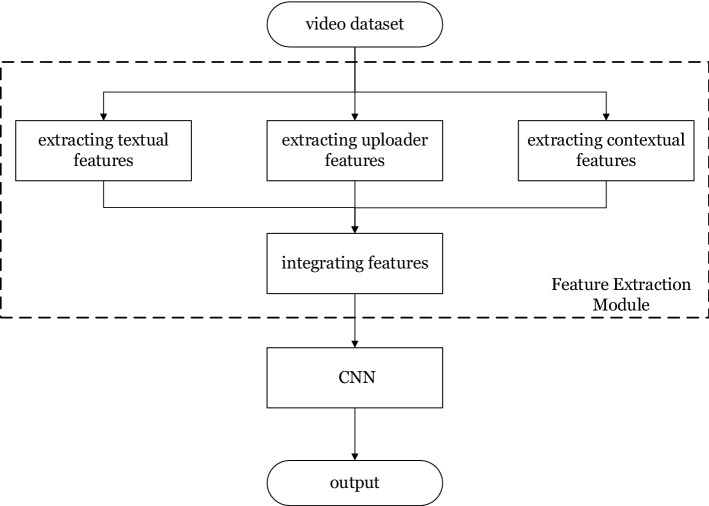
The CNN-based misleading video detection model.

The CNN-based model works to extract and integrate the features of a video, and to detect falsehood of the video by leveraging the CNN, as shown in Fig. 2.

The first part of the proposed model is to extract 16 features under the proposed three categories to obtain a feature set ${{\varvec{a}}}^{({\varvec{n}})}=({{{\varvec{a}}}_{1}}^{\left({\varvec{n}}\right)},{{{\varvec{a}}}_{2}}^{\left({\varvec{n}}\right)},{{{\varvec{a}}}_{3}}^{\left({\varvec{n}}\right)}\dots ,{{{\varvec{a}}}_{16}}^{\left({\varvec{n}}\right)})$ for each sample, where n denotes the n-th sample; then the k-th feature of the dataset is denoted as ${{\varvec{a}}}_{{\varvec{k}}}=({{\varvec{a}}}_{{\varvec{k}}}^{\left(1\right)},{{\varvec{a}}}_{{\varvec{k}}}^{\left(2\right)},{{\varvec{a}}}_{{\varvec{k}}}^{\left(3\right)},\dots ,{{\varvec{a}}}_{{\varvec{k}}}^{\left(4\right)})$ and the average value of the feature in the dataset is ${{\varvec{A}}{\varvec{V}}{\varvec{G}}}_{{\varvec{k}}}$. The feature values for each sample are then normalized as follows:

7 $$\begin{array}{c}{{\varvec{a}}}_{{\varvec{k}}}^{{{\prime}}\left({\varvec{n}}\right)}=\frac{{{\varvec{a}}}_{{\varvec{k}}}^{\left({\varvec{n}}\right)}-{\varvec{min}}\left({{\varvec{a}}}_{{\varvec{k}}}\right)}{{\varvec{max}}\left({{\varvec{a}}}_{{\varvec{k}}}\right)-{\varvec{min}}\left({{\varvec{a}}}_{{\varvec{k}}}\right)}\end{array}$$

Each sample is integrated into a 16 × 16 two-dimensional feature set ${{\varvec{M}}}^{({\varvec{n}})}$:

1. If $${\varvec{i}}={\varvec{j}}$$:
  8 $$\begin{array}{c}{{\varvec{M}}}_{{\varvec{i}}{\varvec{j}}}^{\left({\varvec{n}}\right)}={{\varvec{a}}}_{{\varvec{i}}}^{{{\prime}}\left({\varvec{n}}\right)}\end{array}$$
2. If $${\varvec{i}}<{\varvec{j}}$$:
  9 $$\begin{array}{c}{{\varvec{M}}}_{{\varvec{i}}{\varvec{j}}}^{\left({\varvec{n}}\right)}={{\varvec{a}}}_{{\varvec{i}}}^{{{\prime}}\left({\varvec{n}}\right)}{{\varvec{a}}}_{{\varvec{j}}}^{{{\prime}}\left({\varvec{n}}\right)}\times \frac{{{\varvec{a}}}_{{\varvec{i}}}^{{{\prime}}\left({\varvec{n}}\right)}-{{\varvec{A}}{\varvec{V}}{\varvec{G}}}_{{\varvec{i}}}}{\left|{{\varvec{a}}}_{{\varvec{i}}}^{{{\prime}}\left({\varvec{n}}\right)}-{{\varvec{A}}{\varvec{V}}{\varvec{G}}}_{{\varvec{i}}}\right|}\end{array}$$
3. If $${\varvec{i}}>{\varvec{j}}$$:
  10 $$\begin{array}{c}{{\varvec{M}}}_{{\varvec{i}}{\varvec{j}}}^{\left({\varvec{n}}\right)}={{\varvec{a}}}_{{\varvec{i}}}^{{{\prime}}\left({\varvec{n}}\right)}{{\varvec{a}}}_{{\varvec{j}}}^{{{\prime}}\left({\varvec{n}}\right)}\times \frac{{{\varvec{a}}}_{{\varvec{j}}}^{{{\prime}}\left({\varvec{n}}\right)}-{{\varvec{A}}{\varvec{V}}{\varvec{G}}}_{{\varvec{j}}}}{\left|{{\varvec{a}}}_{{\varvec{j}}}^{{{\prime}}\left({\varvec{n}}\right)}-{{\varvec{A}}{\varvec{V}}{\varvec{G}}}_{{\varvec{j}}}\right|}\end{array}$$

The three categories of features are integrated into a two-dimensional feature set and sent to the convolutional neural network for training. The receptive field of the convolutional neural network is used to explore the potential connections between each features to identify misleading videos.

**Figure 3:**
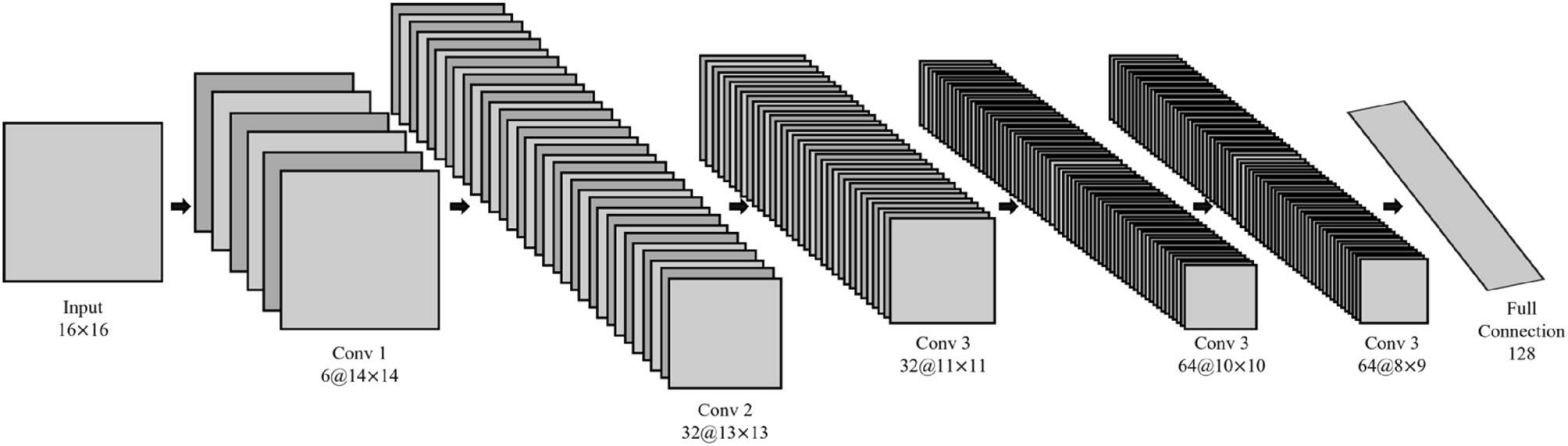
A convolutional neural network (CNN).

Figure 3 shows the structure of a CNN. The neural network comprises six layers, five of which are convolutional layers and one fully connected layer. The convolutional kernels are 3 × 3, 2 × 2, 3 × 3, 2 × 2 and 3 × 3, and all are ReLU activation functions, except for the first convolutional layer, which uses the tanh activation function. As the input feature matrix of the network is a small matrix, the model structure is not used for dimensionality reduction of the pooling layer, but for direct layer-by-layer convolutional feature extraction and feature learning.

## Experiment and analysis

This section presents the experimental results generated by our CNN-based misleading video detection model, and the analysis of these outcomes. Comparisons between our proposed model and some other common machine learning models is also presented here.

### Dataset

The data used for the study are health-related videos collected from Bilibili, a popular video sharing platform in China. The misleading videos are correct for each footage (e.g., alcohol can disinfect, and wine is rich in alcohol). However, the conclusion of the whole video is incorrect after grafting (e.g., alcohol can prevent disease). A web spider was designed to crawl data from the website. The variables of the dataset include video identifier, URL, video title, textual content, view count number, video length, etc.

Ten medical experts were invited to judge the collected videos. All medical experts have doctoral degrees and more than three years of clinical experience. All experts were asked to judge each video independently. For videos that have achieved a high degree of agreement among the experts (> 70%), we directly determined their truth through majority votes. For the remaining controversial videos, experts had a discussion to determine whether the video is real or misleading. Videos whose authenticity or falsity could not be determined by experts after the discussion were deleted from the dataset. The dataset initially contained 867 videos, and 187 that could not be judged real or false were deleted after evaluation by experts. As a result, our final data set contains 700 videos, of which 490 are real videos and 210 are false.

### Experimental results and analysis

Table 1 shows the features extracted from the dataset. In the study, Jieba, a Chinese word segmentation tool, was employed to preprocess the texts in the dataset, including the removal of particles and stop words. The sentiment lexicon of Hownet, including the Chinese-English dictionaries of qualifiers and evaluative and emotive words, was used for sentiment analysis of texts. Moreover, the baseline for the latest upload was set on January 1, 2021, with those released before the time shown as negative values, and otherwise positive. Also, the date of the latest upload was not specified for the to-be-detected videos. Should an uploader post such a video, we would consider January 1, 1900, as the date of the latest upload, meaning that the newest video of the uploader was posted 44,197 days before.

**Table 1:** Features of the misleading video dataset.

Feature category		Mean	S.D	Min	Median	Max	N
Content features	C-Sen-Po	20.07	23.93	− 17	13	202.6	700
	C-Num-MoPar	23.09	28.07	0	12	209	700
	C-Num-PerPro	− 1.36	10.40	− 64	0	53	700
	C-Vtext-Len	706.90	634.76	26	475	3779	700
Uploader features	FF-R	6754.39	41,781.37	0	38.5	584,000	700
	Num-Likes	117,549.29	748,769.63	0	237.5	16,265,714	700
	Re-upload	80.51	158.05	− 1743	115	181	700
	Num-ToVi	3,914,180.37	54,192,132.06	0	28,500	1,257,389,046	700
Environment features	Likes	184.48	–	0	–	27,000	700
	Retweets	29.84	–	0	–	3084	700
	Favourites	46.87	–	0	–	3982	700
	Rewards	22.75	–	0	–	5111	700
	E-Sen-Po	0.78	3.09	− 8	0	28	700
	E-Num-MoPar	1.16	2.94	0	0	25	700
	E-Num-PerPro	0.18	1.21	− 15	0	7	700
	E-Vtext-Len	32.50	85.68	0	0	771	700

**Figure 4:**
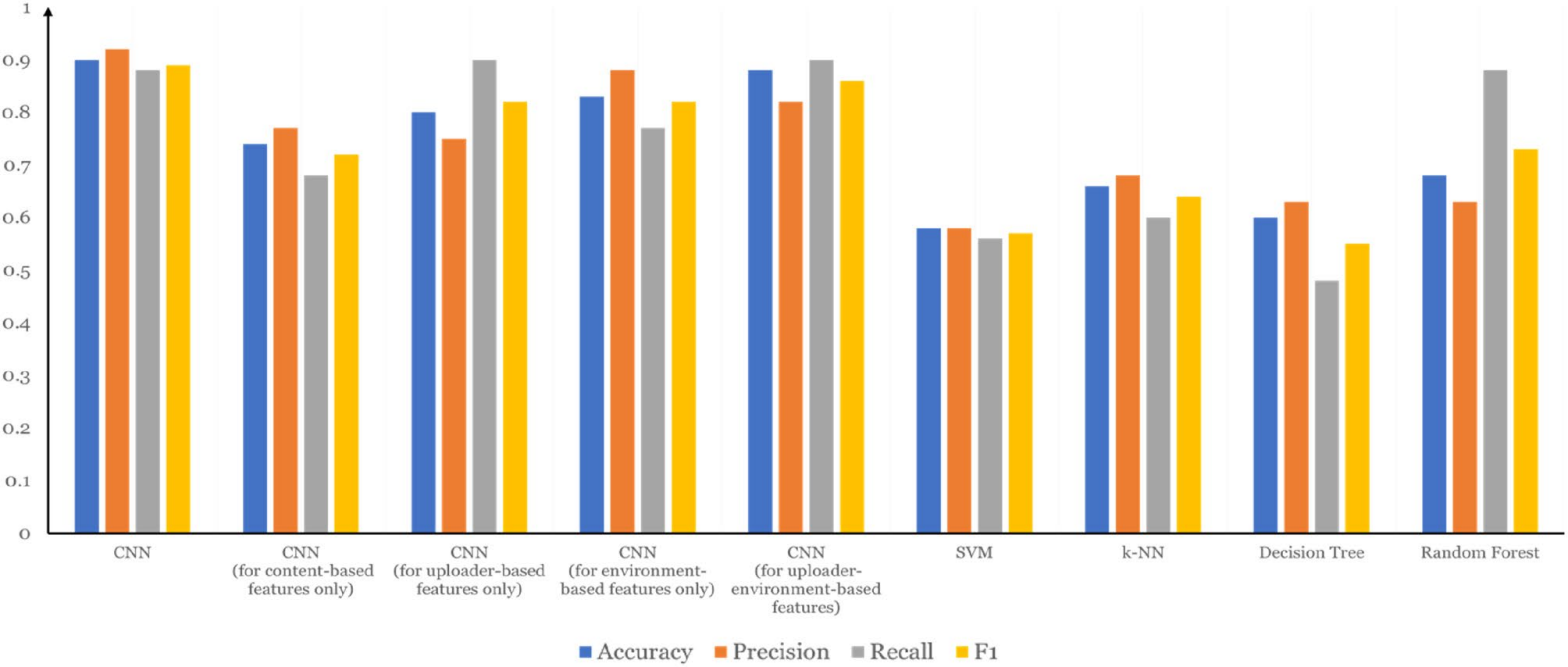
Experiment results from different models.

We used the 16 features of the dataset to create a 16 × 16 feature matrix, with the eigenvalue of the 16 features lying on the principal diagonal. The matrix was then imported into a CNN for training. Given that the dataset involved only 700 pieces of data, which is a small pool of samples, the input for training of the neural network was randomly picked, and each time we picked 200 videos, among which 100 were authentic health-related clips and 100 were misleading videos. When it came to the detection dataset, 60 videos, in which 30 were authentic and the rest misleading, were picked randomly and different from those for training. The experimental results, the experiments on feature variety ablation for the models in this paper, and a comparison of the experimental results for the four machine learning techniques (support vector machines^38^, KNN^39^, decision trees^40^ and random forests^41^) are shown in Table 2 and Fig. 4.

**Table 2:** Comparison of different detection models.

Misleading video detection models	Accuracy	Precision	Recall	F1
CNN	0.90	0.92	0.88	0.89
CNN (for content-based features only)	0.74	0.77	0.68	0.72
CNN (for uploader-based features only)	0.80	0.75	0.9	0.82
CNN (for environment-based features only)	0.83	0.88	0.77	0.82
CNN (for upload-environment-based features)	0.88	0.82	0.9	0.86
SVM	0.58	0.58	0.56	0.57
k-NN	0.66	0.68	0.60	0.64
Decision tree	0.60	0.63	0.48	0.55
Random forest	0.68	0.63	0.88	0.73

As shown in Fig. 4, the proposed method in this study (marked by CNN in Fig. 4) achieved the best performance in general, with an accuracy of 90%. Specifically, the proposed method achieved a precision of 0.92, which is high and remarkable. The results in Fig. 4 suggest the importance of combining all three categories of features because models that adopt only content features, uploader features or environment features achieved an F1 score of 0.72, 0.73, and 0.73, respectively, which are all lower than the F1 score achieved by our model that combines the three categories of features (0.84). The figure also shows that the proposed CNN model achieved a higher F1 score than SVM (0.57), k-NN (0.64), decision tree (0.55), and random forest (0.73).

## Conclusions

The paper proposes a CNN-based model that could effectively detect misleading videos by considering three categories of features (i.e., content features, uploader features and environment features). Among the three categories of features, the environment features are proposed in this research for the first time. The experiments showed that all three categories of features play vital roles in detecting misleading videos. Compared with models that consider only one or two feature categories, our approach that combines content features, uploader features and environment features achieved the best performance in general. In addition, the proposed CNN-based approach outperformed other baselines such as SVM, k-NN, decision tree and random forest. This finding suggests that deep learning approaches are more appropriate for misleading video detection than other methods. Although the misleading video detection method in this study is proposed based on Bilibili, it is a general framework which should also be applicable to other video sharing websites such as YouTube and TikTok.
